# Concrete Modular Pavement Structures with Optimized Thickness Based on Characteristics of High Performance Concrete Mixtures with Fibers and Silica Fume

**DOI:** 10.3390/ma14123423

**Published:** 2021-06-21

**Authors:** Audrius Vaitkus, Judita Gražulytė, Ovidijus Šernas, Martynas Karbočius, Rafal Mickevič

**Affiliations:** Road Research Institute, Vilnius Gediminas Technical University, 08217 Vilnius, Lithuania; audrius.vaitkus@vilniustech.lt (A.V.); ovidijus.sernas@vilniustech.lt (O.Š.); martynas.karbocius@vinliustech.lt (M.K.); rafal.mickevic@vilniustech.lt (R.M.)

**Keywords:** high-performance concrete, slab, pavement, modular pavement, pavement design, precast concrete pavement (PCP), tensile splitting strength, flexural strength

## Abstract

Usually, C30/37 strength class concrete is used to construct concrete pavements on a rigid, semi-rigid or flexible base. Concrete with such a strength delivers essential design characteristics: flexural strength and tensile splitting strength are between 4.5–5.4 MPa and 2.8–3.7 MPa, respectively. Design characteristics can be significantly increased by densifying the concrete mixture, i.e., adding silica fume, steel or polypropylene macro fibers. As high-performance concrete characteristics are 20–60% higher than those for standard concrete (C30/37), new possibilities to reduce the thickness of concrete pavement slabs appear. The theoretical analysis of concrete pavement structures with high-performance concrete mixtures (C40/50, C45/55 and C50/60) showed that slab thickness could be reduced by 6–39% compared to a standard concrete pavement structure depending on the concrete properties and design method. From all those pavement structures, three concrete mixtures were determined as the most rational ones in terms of PCP thickness reduction and total pavement cost: (i) with 49.5 kg/m^3^ of steel fibers and 25.2 kg/m^3^ of silica fume; (ii) with 10.0 kg/m^3^ of polypropylene fibers (type A); (iii) with 49.5 kg/m^3^ of steel fibers.

## 1. Introduction

The operation and maintenance of a road network is a challenge, and it becomes even more complicated with the continuous growth of traffic. It is estimated that passenger transport will grow by up to 42% by 2050 and freight transport up to 60% [1]. For such heavily loaded roads, concrete pavements become superior to asphalt pavements.

Typically, a concrete mixture with a strength class of C30/37 is used to construct concrete pavements. In these cases, the pavement thickness varies from 175 to 400 mm depending on the traffic, environmental conditions, base type (rigid, semi-rigid or flexible), slab dimensions and concrete mixture characteristics [2,3,4,5]. Pavement thickness is a crucial aspect in pavement design, however, a compromise between pavement performance and construction cost often has to be reached. It is significantly important for concrete modular pavements, also known as precast concrete pavements (PCPs), since the thickness affects the transportation and lifting cost of slabs and, at the same time, the whole construction cost. It is noted that those pavements differ from other types of concrete pavements by production (construction) process: PCPs are constructed of prefabricated slabs that are transported to the project site from the plant only when the desired concrete strength is achieved, while other types of concrete pavements are cast-in-place and the concrete mixture cures on-site [6]. PCPs, like other concrete pavement types, are suitable for all types of application areas, i.e., roads, streets, aprons and taxiways, logistic terminals, low volume and private roads, as well as bicycle and pedestrian paths [7].

One of the options to reduce the thickness of the slab is to improve the design characteristics of the concrete mixture. It is achieved by densifying the mixture, i.e., adding silica fume, steel or polypropylene macro fibers into the concrete mixture. Mostly, the purpose of these admixtures is to improve compressive strength due to hydraulic or pozzolanic (or even both) activity in high-strength concrete. Admixtures can be added as supplementary components or just as a replacement for cement. Compared to fly ash, natural zeolite or ground granulated blast, silica fume is exceptional [8,9]. Frequently, studies have used silica fume in the amount of 5–10% by mass of cement in concrete [8,9,10]. Nonetheless, the amount of silica fume can be boosted up to 15%, sometimes even up to 20–30% [11,12,13,14]. Depending on the replaced amount of cement, the addition of silica fume increases compressive strength after 28 days by 10–40% compared to a reference specimen. Moreover, silica fume improves the modulus of elasticity by 10–45%, indirect tensile strength by 17–23% and flexural strength by 7–64% [8,10,11,13,15]. The resistance to fatigue of mixtures with silica fume is barely investigated. However, Yan et al. [14] analyzed the effect of cement replacement with silica fume on concrete fatigue resistance. The research results indicated (after a million loads applied) a 38% improvement of fatigue strength compared with a reference mixture, which was noted, leads to shorter and a lower number of cracks. Despite the advantages of silica fume, fibers also have a great impact on concrete characteristics. Fibers can be made of synthetic (polypropylene) or natural materials (steel, glass). Furthermore, fibers are contrasting in terms of length (6–150 mm), thickness (0.005–0.75 mm) and shape (round, deformed, flat, crimped). Due to sufficient structural performance and low cost, the most generally used fibers are steel and polypropylene. Furthermore, steel fibers have hooks at the ends, which creates a resistance to pullout and improves flexural strength and energy absorption capacity compared with straight fibers [16]. The usage of fiber in compressive strength causes only 3–11% higher results [17,18,19,20]. Nonetheless, fibers have an important influence on indirect tensile strength and flexural strength. Indirect tensile strength improves 13–133%, and flexural strength improves 14–120%. The gap between values is formed because of fiber type, length (12–60 mm), shape (hooked-ended, straight or fascicular staple) and of course, amount (0.1–3.0%) [20,21,22,23,24]. Studies have been carried out that show fibers to decrease deflection, crack width and number of cracks and improve load capacity; all of these lead to extend the concrete’s service life [14,17,24,25,26]. Fibers give ductility to concrete, connect microcracks, which appear in materials after cycling loads, and improve concrete’s service life. In the case of advanced binding and improved fatigue life, a fibers direction is recommended to be orientated perpendicular to the loading axis [19]. Above all, the most important note is that fiber content and aspect ratio are more important to resistance to fatigue than the fiber’s orientation and type [24,27,28].

Cajka et al. (2020) [29] analyzed the performance of four slabs with different amounts of fibers (0–75 kg/m^3^) constructed on the subsoil. The study showed that fibers significantly increase the total load capacity of the slab and that load capacity increases further with the increase in fiber content. Higher content of fibers also significantly influenced the total deformations, which are important for the serviceability limit state. In general, an improvement of concrete characteristics leads to a reduction in the thickness of concrete slabs [30]. Parker [31] found that fiber reinforced concrete reduces the thickness of the thin concrete layer by 30% to 50%. Vaitkus et al. (2019) [32] determined that an increase in tensile splitting strength from 3.7 (C30/37) to 4.8 MPa (C40/50) leads to a 15.5% thinner PCP slab. They also concluded that the minimum thickness of PCP slab, produced from concrete mixture C40/50, is 218 mm. In this case, the slab’s width is 3.5 m and length either 4.2 m or 4.4 m [32]. All these facts support the idea that fiber reinforced concrete is becoming a high-tech material that provides excellent performance but requires competent design [33]. Rodden et al. (2019) [34] have made a comparison between the calculated PCP slab thickness with design software StreetPave, OptiPave, WinPas and Pavement ME. The biggest difference in PCP slab thickness was observed when traffic volume was low. Calculations with increased traffic volume have shown smaller differences between different design software.

Taken together, the effect of concrete mixture composition (e.g., silica fume, steel or polypropylene macro fibers) on concrete performance is now well established. However, only a few studies have investigated how the improved design characteristics influence the pavement thickness and how it differs depending on the design method. The purpose of this paper is to identify the most rational pavement structures with high-performance concrete mixtures (C40/50, C45/55 and C50/60) in terms of PCP thickness and total pavement cost and to show the benefit of concrete mixtures with enhanced design characteristics compared to a standard concrete mixture. Since there exist a number of different pavement design methods and it is unclear which one should be used and how the results differ among them, the effect of different calculation methods on slab thickness was analyzed in this paper as well. All pavement structures were designed to withstand 76 million equivalent single axle loads for 30 years.

## 2. Effect of Concrete Characteristics on Pavement Performance

Depending on the design methodology, different mechanical characteristics of concrete are used to calculate the required PCP thickness. Designing concrete pavement structures according to Richtlinien für die rechnerische Dimensionierung von Betondecken im Oberbau von Verkehrsflächen RDO Beton 09 [35], the two main characteristics—tensile elastic modulus and tensile splitting strength—are used. The tensile elastic modulus is related to the compressive elastic modulus, which is recalculated from the compressive strength. In the RDO Beton 09 methodology, it is assumed that the tensile elastic modulus is approximately 1.15 times larger than the compressive elastic modulus. It was observed that the results are more affected by the tensile splitting strength as it directly affects the strength of pavement, while tensile elastic modulus is incorporated in the calculation of pavement response to loads [32]. The coefficient of thermal expansion is also used in the calculations to estimate the bending moment caused by the temperature regime. However, this method does not require an accurate determination of this characteristic or verification of the guide values given in the methodology. In general, the coefficient of thermal expansion has little effect on the thickness of the concrete pavement since the bending moment caused by the temperature regime is not decisive for the performance of the pavement structure under normal conditions. 

In contrast, when using design software such as StreetPave or FAARFIELD, the most important mechanical characteristic of concrete is flexural strength. Design software FAARFIELD and StreetPave use similar concrete mechanical characteristics of concrete in calculations. The main difference between these two design software is that FAARFIELD is fully adapted for airfield pavement calculations. FAARFIELD is using a different load spectrum, which is expressed by different types of aircraft. The FAARFIELD design process currently considers only one mode of failure for rigid pavement: bottom-up cracking of the PCP slab. Cracking is controlled by limiting the horizontal stress at the bottom of the PCP slab and does not consider the failure of subbase and subgrade layers. A three-dimensional finite element model is used to compute the edge stresses in PCP slabs. The model has the advantage of considering where the critical stresses for slab design occur. Critical stresses normally occur at slab edges but may be located at the center of the slab with certain aircraft gear configurations [36].

Design program StreetPave calculations are based on the mechanistic–empirical pavement design method. The design methodology used in StreetPave was taken from the PCA’s Thickness Design for Concrete Highways and Streets manual [37]. The procedure incorporates mechanistic components (load/stress/deflection) with empirical observations, including results from the American Association of State Highway and Transportation Officials Road Test, to establish a thickness design [38]. The analysis procedure contains two separate components: fatigue and erosion. The fatigue analysis simply evaluates the fatigue of the PCP slab at mid-slab at the edge of the pavement. According to the design methodology used in StreetPave, the fatigue analysis procedure estimates fatigue damage using Miner’s damage model. The use of Miner’s fatigue damage model allows for total cumulative fatigue damage to be estimated for all axle types and loads of varying magnitudes because the Miner’s damage model allows for the linear accumulation or summing of fatigue damage. Fatigue damage is defined as the ratio of the number of actual load applications divided by the number of allowable applications to failure. Although the number of actual load applications is determined using statistical forecasting methods and estimates/counts of past traffic, the number of allowable applications to failure is determined on the basis of the ratio of applied equivalent stress (caused by traffic loading) to PCP flexural strength [39]. The erosion analysis evaluates the potential for a concrete pavement to fail by pumping, erosion of the foundation support and/or joint faulting and is based on corner deflections.

The other known software for calculation PCP thickness is MnDOT’s RigidPave design software. This software is based on the 1981 AASHTO Interim Guide. Under this design method, MnDOT designs and constructs only jointed plain concrete pavement (JPCP). Slab thickness is determined using the cumulative 35-year design-lane concrete equivalent single axle loads (CESALs), which are based on the AASHTO load equivalency factors (LEFs). The equation was developed from the AASHTO road test and solves for the cumulative number of ESALs a pavement can withstand before it falls to a given serviceability level. The values used in RigidPave calculations are terminal serviceability, modulus of subgrade reaction, concrete modulus of rupture, concrete modulus of elasticity and number of ESALs to reach terminal serviceability [38].

The method, detailed in mechanistic–empirical pavement design guide, in addition to flexural strength, also includes the modulus of elasticity and the coefficient of thermal expansion. It was observed that crack rate prediction is the least sensitive to changes in flexural strength but is most sensitive to changes in coefficient of thermal expansion [5]. In this case, there is a certain contrast with the RDO Beton 09 method, in which, as mentioned above, the coefficient of temperature expansion does not play a decisive role.

Overall, major characteristics affecting PCP thickness are tensile splitting strength, tensile elastic modulus and flexural strength.

## 3. Analytical Calculations of PCP Thickness

Two methods with different methodological principles and required mechanical characteristics of the concrete were chosen to calculate the PCP thickness. Since mechanistic-empirical methods are more widely used for the design of pavement structures, the open-access software StreetPave (The American Concrete Pavement Association, Washington, D.C., the USA), was selected, which complies with the principles of mechanistic-empirical design methods. Moreover, Rodden et al. (2019) [34] stated that the design software StreetPave provides the most accurate calculations compared to the other design software such as OptiPave, WinPas or Pavement ME. Another chosen method, detailed in the guide RDO Beton 09, is based on semi-probabilistic analysis and is inherently more attributable to empirical methods.

### 3.1. Methods and Input Data for Calculations

PCP thickness was calculated for the service life of 30 years irrespective of the used method. In all cases, the number of equivalent single axle load (10 t) was 76 million. The required PCP thickness was calculated for structures with different base layers—unbound crushed aggregate base and hydraulically bound base layer. The reinforcement of the joints was also included as a variable—calculation was made assuming that the reinforcement was provided by dowels in transverse joints and reinforced anchors in longitudinal joints as well as without reinforcement at all.

The mechanical characteristics of high-performance concrete mixtures used to calculate the PCP thickness according to both methods are presented in Table 1. At least three specimens were tested to determine each characteristic. The data in Table 1 are given as an average of those values. The individual values and methods of how these characteristics were determined are given in [40], also explained in detail is the composition of those mixtures and the difference between polypropylene fiber A (Fibrocev, Sirone, Italy) and B (Adfil N.V., Zele, Belgium).

When using the method detailed in RDO Beton 09, the slab thickness was calculated corresponding to the boundary conditions of bearing capacity, serviceability and fatigue resistance (Table 2). The load-bearing boundary condition corresponds to the bending moment that the pavement can withstand before structural damage or failure occurs. The boundary condition of serviceability corresponds to conditions in which, if exceeded, the specified requirements of a structure or part of a structure are no longer met, or a permanent load-bearing capacity is no longer ensured. Fatigue boundary condition corresponds to the permanent stresses occurring in the form of accumulated axle loads. The bending moments are calculated for all boundary conditions at the center of the longitudinal and transversal joint. The basis of concrete pavement design is the assurance that the limit bending moments are no less than the design bending moments in all analysis cases [6]. 

The limit bending moment for each boundary condition is calculated according to Equation (1).

(1) $$M_{R}=0.167\cdot h_{d}^{2}\cdot f_{d}$$

where: $M_{R}$ is the limit bending moment, Nmm/mm; $h_{d}$ is the thickness of the concrete pavement, mm; $f_{d}$ is the calculated concrete strength, based on tensile splitting strength, N/mm^2^.

The design bending moment for each boundary condition is calculated as a sum of design moment M_EV_ caused by traffic load (Equation (2)) and design moment M_ET_ caused by temperature regime (Equation (3)).

(2) $$M_{EV}=m_{bL}\cdot m_{bD}\cdot F_{d}\cdot 1000\left[0.55\log\left(\frac{I_{v}}{b}\right)+0.1\frac{b}{I_{v}}-0.011\right]$$

where: $M_{EV}$ is the design bending moment caused by traffic load, Nmm/mm; $m_{bL}$ is the bedding factor; $m_{bD}$ is the joints reinforcement factor; $F_{d}$ is the adjusted wheel load, kN; $I_{v}$ is the elastic length of concrete slab, mm; b is the radius of the circular contact area, mm.

(3) $$M_{ET}=\alpha_{cT}\cdot \gamma_{tot}\cdot E_{ctm}\frac{h_{d}^{3}\cdot m_{T1}\cdot m_{T2}\cdot m_{T3}\cdot \delta_{t}}{12}$$

where: $M_{ET}$ is the design bending moment caused by temperature regime, Nmm/mm; $\alpha_{cT}$ is the thermal expansion coefficient, 10^−6^/K; $\gamma_{tot}$ is the factor for taking the slow deformation build-up under thermal stress into account; $E_{ctm}$ is the tensile elastic modulus, N/mm^2^; $h_{d}$ is the thickness of the concrete pavement, mm; $m_{T1}$, $m_{T2}$, $m_{T3}$ is the temperature-related adjustment factors; $\delta_{t}$ is the temperature gradient, K/mm.

The essential factor for the calculation of concrete pavement thickness is the characteristic tensile splitting strength of the concrete. In addition to the fact that the limit bending moment depends directly on the characteristic tensile splitting strength, this characteristic is also used to define the probability of failure. The material factor, which determines the probability of failure and its relationship to the road category, is defined by the Gaussian normal distribution of the tensile splitting strength. Concrete pavement is not dimensioned as a part of a package of layers. It is analyzed as a stand-alone component with the provision that concrete pavement works on its own, as a result of its different temperature dependencies in comparison with those of the base courses and the different material parameters such as the elasticity modulus, splitting tensile strength, thermal expansion coefficient as well as their necessary division into individual slabs through joints. The method does not directly include the load-bearing capacity and thickness of the subgrade and subbase layers but is related to the technical regulation requirements of the frost resistance of the structure and the load-bearing characteristics of the individual layers. Only the bearing capacity of the base layer is directly estimated in the calculation. However, it should be noted that the bedding modulus of the hydraulically bound base layer does not require verification, and the same fixed value is used in all cases. This means that the results are only valid if all the requirements of technical regulations for structural strength and resistance to adverse climate factors are met. The input parameters for calculation according to RDO Beton 09 are given in Table 3.

Using the method according to PCA design methodology, the slab thickness was calculated with the automated design software StreetPave. The design inputs are shown in Table 4 and Table 5. The thickness and E modulus of the subgrade and frost-resistant layer were chosen according to technical regulations KPT SDK 19 [41]. The E modulus of the hydraulically bound base was chosen according to the average value available in the design software StreetPave. Based on the StreetPave library, the hydraulically bound base was assumed as a lean concrete subbase, frost-resistant layer and crushed aggregates base layer—as an unstabilized subbase. From Table 5, it can be seen that, in opposite to RDO Beton 09, StreetPave does not take into account the speed of vehicles, the thermal characteristics of the concrete and the dimensions of the PCP slab. A flexural strength of concrete mixtures, which is used in design with StreetPave, is given in Table 1. It has to be noted that the highest value of flexural strength, which can be used in the design program StreetPave, is 8.27 MPa. Therefore, if a flexural strength of an analyzed concrete mixture was higher than 8.27 MPa, the value was extrapolated. The design criteria used in StreetPave are presented in Table 2.

### 3.2. Calculations with Different Methods

**Figure 1:**
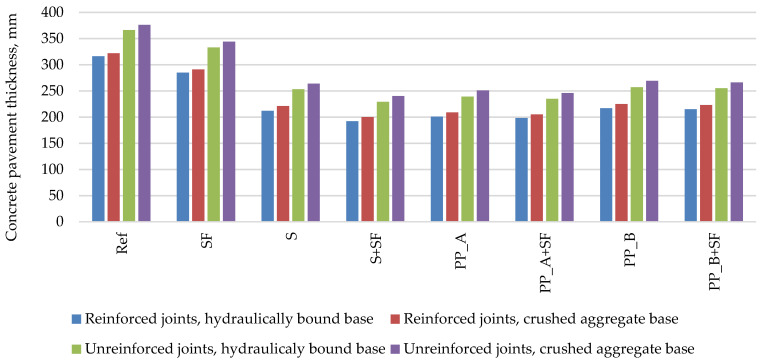
The minimum PCP thickness calculated by the RDO Beton 09 method.

The minimum PCP slab thickness calculated according to RDO Beton 09 is presented in Figure 1. The calculation results show that using a reference concrete mixture without additives, the required pavement thickness is the highest compared to other mixtures and varies from 316 to 376 mm depending on the base layer type and joint reinforcement option. Depending on the type of used additives, the pavement thickness can be reduced by 9% to 39%. It can be seen that the addition of silica fume reduces the thickness by 9–10%; in this particular case of calculation, it means 31–33 mm. The lowest pavement thickness is achieved by using a concrete mixture with steel fiber and silica fume, which varies from 192 to 240 mm depending on the base layer type and joint reinforcement option. Comparing the thickness of the pavement of concrete mixtures with various fibers, it is also clear that the pavement of concrete mixture with steel fiber without silica fume (tensile splitting strength—5.2 MPa) is of a similar thickness as the worst-performing concrete mixture with polypropylene_B fiber (tensile splitting strength—5.0 MPa).

**Figure 2:**
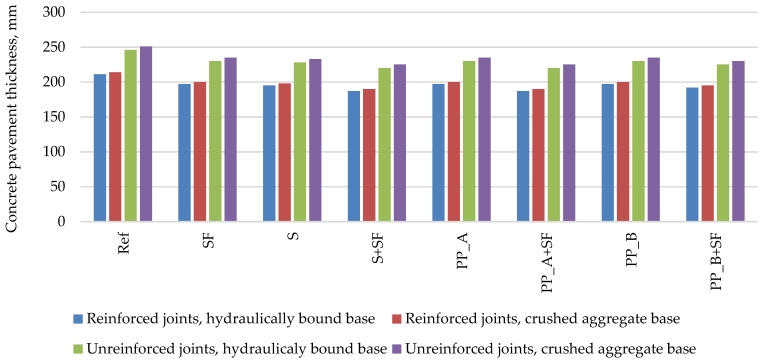
The minimum PCP thickness calculated with the design software StreetPave.

The minimum PCP slab thickness calculated with the design program StreetPave is presented in Figure 2. The calculation results show that using a reference concrete mixture without additives, the required pavement thickness is the highest compared to other mixtures and varies from 211 to 251 mm depending on base layer type and joint reinforcement option. Depending on the additives used, the pavement thickness can be reduced by 6–11%. It can be seen that the addition of silica fume reduces the thickness by 6–7%; in this particular case of calculation, it means 14–16 mm. The lowest pavement thickness is achieved by using a concrete mixture with steel fiber and silica fume and by using a concrete mixture with polypropylene_A fiber and silica fume, which varies from 187 to 225 mm depending on base layer type and joint reinforcement option.

## 4. Analysis and Discussion

**Figure 3:**
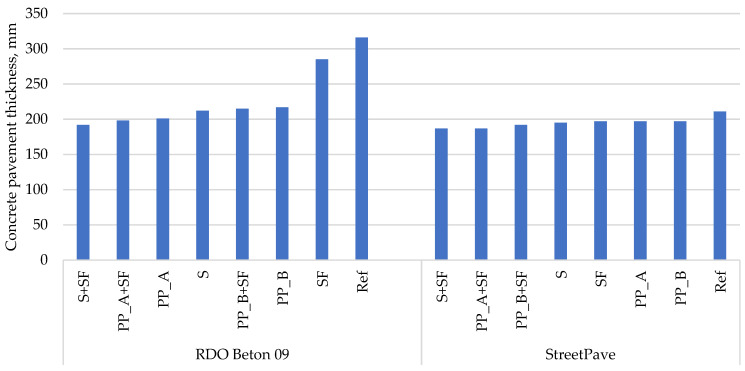
Calculated PCP thicknesses on hydraulically bound base.

**Figure 4:**
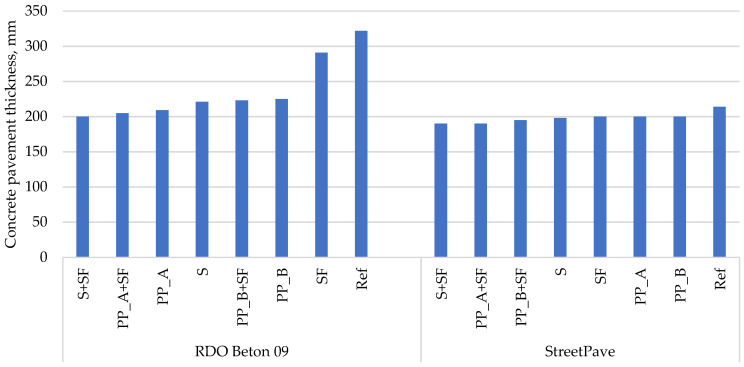
Calculated PCP thicknesses on crushed aggregates base.

Calculations using the RDO Beton 09 method showed that the thickness of the PCP varies from 192 to 366 mm depending on the type of the base layer and the joint reinforcement option (Table 6, Figure 3 and Figure 4). Results showed that the boundary condition of fatigue resistance was decisive in all cases. In terms of bearing capacity and serviceability boundary conditions, the pavement reaches just over 70% and almost 50% of its potential over the same design period, respectively. In all cases, the critical location in the PCP slab was the transverse joint. 

Overall, calculations made by the RDO Beton 09 method showed a more massive concrete pavement thickness required to withstand the same 76 million of equivalent 10 t weight of standard axle load than the design software StreetPave. Calculations made according to RDO Beton 09 showed that concrete pavement with crushed aggregate base and reinforced joints requires a 1.9–4.2% thicker concrete layer than concrete pavement with hydraulically bound base and reinforced joints, with unreinforced joints—from 2.7% to 5%. Calculations made with the design software StreetPave showed that concrete pavement with crushed aggregate base and reinforced joints requires a 1.4–1.6% thicker concrete layer than concrete pavement with hydraulically bound base and reinforced joints, with unreinforced joints—from 2% to 2.3%. Comparison of PCP slab thickness on hydraulically bound base and unreinforced joints with reinforced joints has shown from 15.8% to 19.3% thicker PCP slab according to RDO Beton 09 calculation method and from 16.6% to 17.6% thicker PCP slab according to the design software StreetPave calculation. The comparison of PCP slab thickness on a crushed aggregate base and unreinforced joints with reinforced joints has shown from 16.8% to 20.1% thicker PCP slab according to RDO Beton 09 calculation method and from 17.3% to 18.4% thicker PCP slab according to the design software StreetPave calculation. 

Looking at the obtained results, it is possible to see the interface of the RDO Beton 09 method with the catalog of Lithuanian standard pavement structures. First of all, the RDO Beton 09 method seems to be subject to the condition of equivalence between different base layers. The calculated pavement thickness when using a reference concrete mixture with silica fume (mixture code SF), the mechanical characteristics of which are essentially the same as the German-regulated concrete mixtures for road construction, does not differ significantly when a crushed aggregate base layer or a hydraulically bound base layer is used. In Lithuanian standard pavement structures, regardless of the type of base layer, the thickness of the concrete pavement is constant as the equivalence of different base layers is achieved through its different thicknesses. Furthermore, the PCP thickness calculated according to RDO Beton 09 differs from the Lithuanian standard structure for the same traffic conditions by 5.6–7.8%, which may primarily be related to the large number of variables in the RDO Beton 09 method. Considering this, it can be stated that the method correlates with the catalog of Lithuanian standard pavement structures.

As seen in Figure 3 and Figure 4, the most rational concrete mixtures in terms of PCP thickness reduction are these: (i) with silica fume and steel fibers (S + SF); (ii) with silica fume and polypropylene fibers Type A (PP_A + SF); (iii) with polypropylene fibers Type A (PP_A) or with silica fume and polypropylene fibers Type B (PP_B + SF). The first two concrete mixtures are superior to other mixtures irrespective of the design method. In the case of the RDO Beton 09 method, concrete mixture with silica fume and steel fibers (S + SF) reduces the thickness of PCP slab from 316 to 192 mm on hydraulically bound base course and from 322 to 200 mm on the unbound base course. With the same method, concrete mixture with silica fume and polypropylene fibers Type A (PP_A + SF) reduces the thickness of PCP slab from 316 to 198 mm on hydraulically bound base course and from 322 to 205 mm on the unbound base course. While with StreetPave, both concrete mixtures (S + SF and PP_A + SF) reduce the thickness of PCP slab from 211 to 187 mm on hydraulically bound base course and from 214 to 190 mm on the unbound base course. A third mixture that provides the highest reduction in thickness differs among methods. In the case of RDO Beton 09, concrete mixture with polypropylene fibers Type A (PP_A) is in the third place, while in the case of StreetPave, concrete mixture with silica fume and polypropylene fibers Type B (PP_B + SF) is. It is worth highlighting that the prioritization of concrete mixtures from the thinnest concrete pavement to the thickest one is the same for different types of bases but differs among methods.

The comparison of the findings with those of other studies [30,31,38] confirms that both silica fume and fibers significantly increase the load capacity of the PCP slab and, as a result, reduce its thickness. However, this study showed that the effect of reduction depends not only on the concrete mechanical characteristics, base type and joint type but also on the design method. This phenomenon was also observed by [34]. Further research should be undertaken to determine the most accurate design method. 

**Figure 5:**
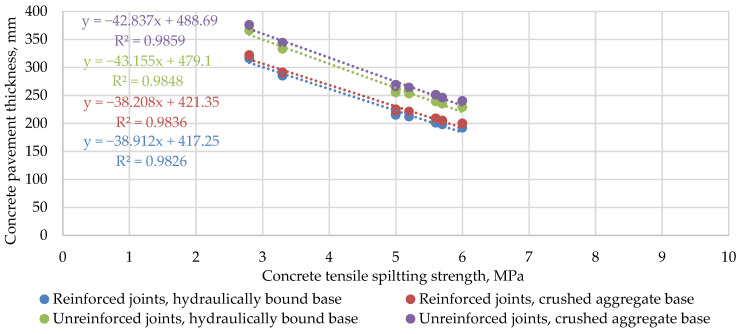
The relationship between the calculated PCP thickness according to the RDO Beton 09 method and the concrete tensile splitting strength.

**Figure 6:**
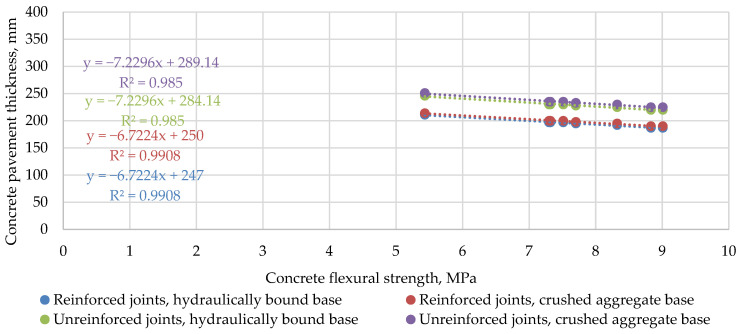
The relationship between the calculated PCP thickness using the software StreetPave and the concrete flexural strength.

Comparing the correlation of concrete pavement thickness with the mechanical characteristics of concrete, it can be seen that the RDO Beton 09 method is significantly more sensitive to the change in the main characteristics of the concrete. As can be seen from Figure 5 and Figure 6, the slope of the trend lines in the case of the RDO Beton 09 method is approximately six times higher compared to the results of the StreetPave software. For example, in the case of the RDO Beton 09 method, when the tensile strength of concrete increases by almost 54%, the thickness of the pavement decreases by 36%. In contrast, the results of the StreetPave calculation showed that an increase in the flexural strength of the same magnitude results in a decrease in pavement thickness of only 9%. Calculations with the design software StreetPave showed that the difference of 0.2 MPa in flexural strength does not affect concrete pavement thickness at all. It was also observed that in the RDO Beton 09 method, the dependence of the calculated thickness on the modulus of elasticity is not so clear, as a higher modulus does not always lead to a lower thickness.

## 5. Cost-Benefit Analysis of High-Performance Concrete

Fiber and/or silica fume reinforced fresh concrete is relatively expensive (especially if the cost of concrete in the plant is used for the cost-benefit analysis), but cost-benefit analysis should reveal the potential benefits of PCP slabs made from concrete with improved characteristics. Each mixture type should be analyzed considering not only economic advantages in materials caused by improvement in the strength and reduced thickness of PCP slab due to the improvement of concrete characteristics but also construction and transportation expenses. For this purpose, the cost of fresh concrete in terms of compressive and flexural strengths, the cost of a square meter of pavement on crushed aggregate base and cost of a square meter of pavement on hydraulically bound base are presented in the following figures. All calculations are theoretical.

**Figure 7:**
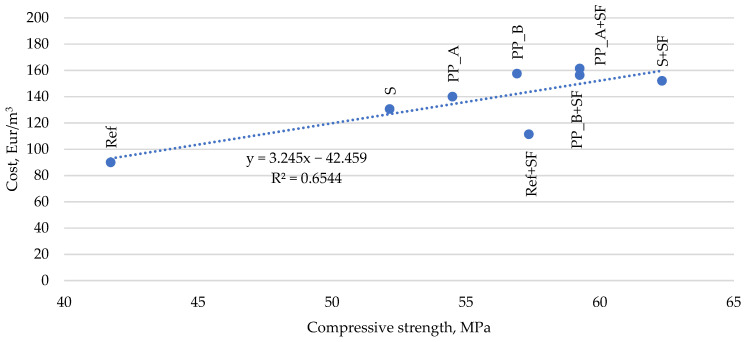
Cost of fresh concrete in terms of compressive strength.

The results of compressive strength indicated the tendency that compressive strength increases with a mixture cost. As expected, reinforced concrete mixtures have higher values than the reference mixture. The mixture with silica fume and steel fibers (S + SF) has the highest values and is even cheaper than mixtures with silica fume and both types of polypropylene fibers (PP_A + SF and PP_B + SF), which does not achieve even 60 MPa. Furthermore, the reference mixture with only silica fume (Ref + SF) showed a high compressive strength value and cost ratio because the mixture achieved 57.3 MPa with EUR 111.42 cost, while mixtures with polypropylene (PP_A and PP_B) did not achieve this compressive strength value and cost even more (Figure 7).

**Figure 8:**
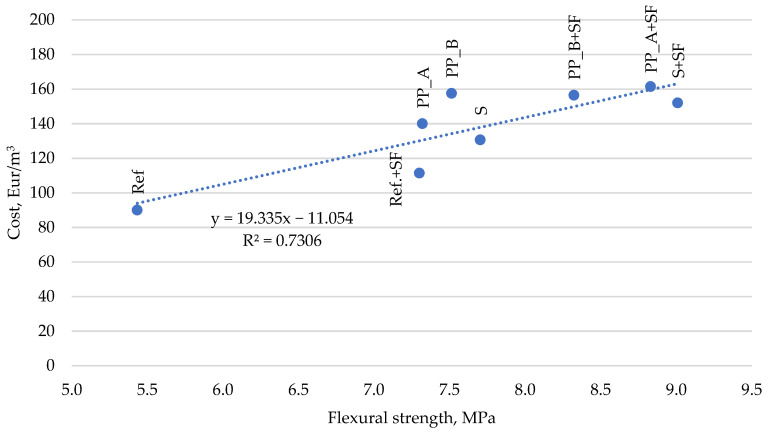
Cost of fresh concrete in terms of flexural strength.

The results of flexural strength and cost ratio indicate that the mixture reinforced with silica fume and steel fiber (S + SF) has the highest values of flexural strength (9.0 MPa), and the cost of this mixture is EUR 152.01. There is no reason to use concrete with silica fume and both types of polypropylenes (PP_A + SF and PP_B + SF) because the mixtures did not achieve as good results as mixtures with silica fume and steel fiber (S + SF), and average costs are even higher. The mixture with only steel fibers (S) also has a satisfying result because it reaches a flexural strength of 7.7 MPa, and the cost is EUR 130.59. Comparing the reference mixture with silica fume (Ref + SF) to the mixture reinforced with steel fibers (S), the reference mixture is an even better choice because it reaches the same flexural strength and cost EUR 111.42 (Figure 8).

The idea of cost-benefit analysis is to evaluate the economic effect of the usage of a thinner PCP slab made from improved concrete instead of using a thicker slab with reference concrete. To evaluate the cost benefit of PCP slabs made from concrete mixtures with improved characteristics, the expenses of fresh concrete mixtures, dowels, slab manufacturing, storage and transportation and PCP pavement construction were considered. It is known that the manufacturing, transportation and installation of thicker slabs require many more expenses compared to thinner slabs. To evaluate the cost of slab manufacturing and installation, the fresh concrete cost was multiplied by a coefficient of 1.15 for the thickest slab and multiplied by a coefficient of 1.0 for the thinnest slab. To evaluate the cost of transportation, the cost of fresh concrete was multiplied by a coefficient of 1.3 for the thickest slab and multiplied by a coefficient of 1.0 for the thinnest slab. The coefficients for other mixtures slabs were interpolated considering the thickness of each PCP slab.

As discussed in previous sections, the StreetPave method is not sensitive to concrete characteristics (maximum difference of calculated PCP slab thickness is only 26 mm) compared to RDO Beton 09 (maximum difference of calculated PCP slab thickness is 137 mm). Even without cost-benefit analysis, it can be seen that the total price of PCP pavement, calculated using StreetPave, would mainly depend on the fresh concrete price. Considering these facts, the cost per square meter of pavement analyses was performed only on results received from RDO Beton 09. 

**Figure 9:**
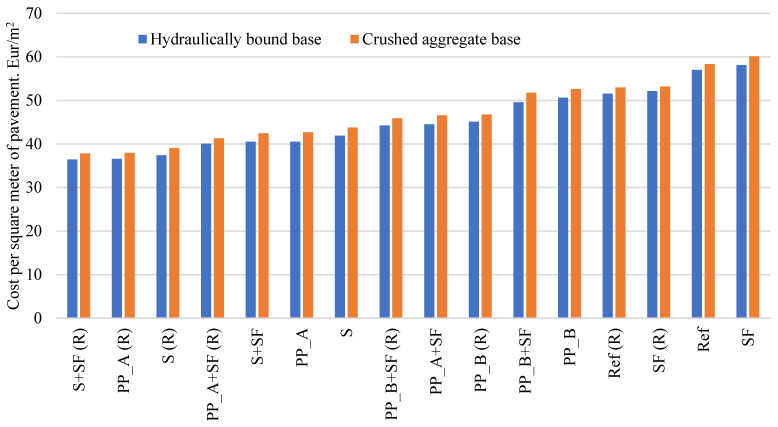
Cost per square meter of pavement on the crushed aggregate base and hydraulically bound base.

Figure 9 represents the results of cost per one square meter of the pavement on a crushed aggregate base and on a hydraulically bound base when the thickness of each pavement is as shown in Table 5. In Figure 9, (R) stands for reinforced joints. The results revealed a significant difference in total PCP pavement cost when comparing pavement with the reference concrete mixture and pavement with improved concrete (with silica fume and/or fibers). In all cases, pavements with improved concrete (with both silica fume and fiber) have lower costs than pavements with the reference mixture or reference concrete with only silica fume. PCP pavements made from S+ SF (R), PP_A (R) and S (R) concrete mixtures with reinforced joints on crushed aggregate base are from 33.1% to 35.2% cheaper compared to the pavement from the reference concrete mixture, while pavement from the SF concrete mixture is 3.1% more expensive compared to the reference mixture. Comparing all PCP pavements, SF and reference have the highest cost (60.14 and 58.33 EUR/m^2^ respectively), while S + SF (R) has the lowest price (37.80 EUR/m^2^). This analysis confirms that the most important factor in cost-benefit analysis is the total cost of PCP construction, not only cost of fresh concrete.

As can be seen from Figure 9, in all cases, pavements on a hydraulically bound base with improved concrete (with both silica fume and fiber) have a lower cost than pavements with the reference mixture or reference concrete with only silica fume. PCP pavements made from S + SF (R), PP_A (R) and S (R) concrete mixtures with reinforced joints on a hydraulically bound base are from 34.4% to 36.2% cheaper compared to the pavement from the reference concrete mixture, while the pavement from the SF concrete mixture is 1.9% more expensive compared to the reference mixture. Comparing all pavements, SF and the reference concrete mixtures have the highest costs (58.11 and 57.02 EUR/m^2^, respectively) while S + SF (R) has the lowest price (36.40 EUR/m^2^).

Cost to benefit analysis shows that the use of concrete with steel fiber or polypropylene A fiber w/ or w/o silica fume and the use of dowels for jointing slabs provides the highest economic effect. Moreover, the use of the reference mixture or SF mixture without fibers for PCP pavement is the most uneconomic.

## 6. Conclusions

The concrete pavement thickness calculations using two different methods, two different base types, reinforced joints and unreinforced joints, as well as a cost-benefits analysis of high-performance concrete led to the following conclusions:

- A minimum thickness of PCP slab depends not only on the pavement structure, joint type and materials characteristics but also on the applied design method. At the same conditions, the semi-probabilistic empirical pavement design method (RDO Beton 09 method) led to a 2–50% thicker PCP slab than the software StreetPave (mechanistic–empirical pavement design method). The lowest difference was determined when a concrete mixture with polypropylene_A fiber and silica fume was used, while a reference mixture gave the highest difference irrespective of the presence or absence of silica fume. The main reason for that is the use of different concrete characteristics for PCP thickness calculation (tensile elastic modulus and tensile splitting strength is required for RDO Beton 09, while StreetPave uses flexural strength) and the different sensitivity to the change in those characteristics.
- The comparison of PCP slab thickness with concrete mechanical characteristics used to calculate the thickness showed that the RDO Beton 09 method is significantly more sensitive to the change in the characteristic values of the concrete than StreetPave software. In the case of the RDO Beton 09 method, the 54% increase in the tensile splitting strength of concrete reduced the thickness of the PCP slab by 36%, while the same percentage increase in flexural strength (StreetPave) reduced the thickness of PCP only by 9%.
- The replacement of standard (C30/37) concrete mixture with a high-performance one (C40/50, C45/55 and C50/60) reduces the thickness of PCP slab from 6% to 39% depending on the design method. With both design methods, the highest decrease in thickness of PCP slab (124 mm with RDO Beton 09 method and 24 mm with StreetPave) was determined when a concrete mixture with tensile splitting strength of 6 MPa, tensile elastic modulus of 91 GPa and flexural strength of 9 MPa was used. Those characteristics were achieved by adding 25.2 kg/m^3^ of silica fume and 49.5 kg/m^3^ of steel fibers in the concrete mixture.
- Joint type has a much larger effect on the PCP slab thickness than base type, irrespective of design method and the mechanical characteristics of the concrete mixture. The thickness of PCP slab with dowel reinforced joints was 14–17% lower than that with unreinforced joints, while hydraulically bound base course resulted in only 2–5% lower thickness than unbound base course (crushed aggregate).
- The cost-benefit analysis showed that the use of a concrete mixture with either steel or polypropylene fibers, irrespective of the presence of silica fume, has a significant economic effect on the total cost per square meter of PCP. Comparing to reference concrete, the total cost per square meter of PCP with fibers decreased from 8.8% to 27.2% when the pavement is on a crushed aggregate base and from 11.2% to 29.0% when the pavement is on a hydraulically bound base. If dowel reinforced joints are used, the economic effect is even higher—the total cost per square meter of PCP decreases from 19.8% to 35.2% when the pavement is on a crushed aggregate base, and from 20.9% to 36.2% when the pavement is on a hydraulically bound base. Based on the theoretical calculations, the use of concrete with both steel fiber and silica fume and reinforced joints is the most economic (37.80 EUR/m^2^) when comparing to the reference mixture (58.33 EUR/m^2^).
- From all analyzed pavement structures with different concrete mixtures, four concrete mixtures, which are listed below, can be assumed as the most rational ones in terms of PCP thickness reduction and total pavement cost:
  − Concrete mixture with 49.5 kg/m^3^ of steel fibers and 25.2 kg/m^3^ of silica fume (reduces the thickness of PCP slab from 316 to 192 mm on hydraulically bound base course and from 322 to 200 mm on the unbound base course);
  − Concrete mixture with 10.0 kg/m^3^ of polypropylene fibers (type A) (reduces the thickness of PCP slab from 316 to 201 mm on hydraulically bound base course and from 322 to 209 mm on the unbound base course);
  − Concrete mixture with 49.5 kg/m^3^ of steel fibers (reduces the thickness of PCP slab from 316 to 212 mm on hydraulically bound base course and from 322 to 221 mm on the unbound base course);
  − Concrete mixture with 10.0 kg/m^3^ of polypropylene fibers (type A) and 25.2 kg/m^3^ of silica fume 49.5 kg/m^3^ (reduces the thickness of PCP slab from 316 to 198 mm on hydraulically bound base course and from 322 to 205 mm on the unbound base course).

Based on the theoretical calculations, a lower cost than 40 EUR/m^2^, including the expenses of fresh concrete mixture, dowels (if needed), slab manufacturing, storage and transportation, is achieved if PCP with reinforced joints is constructed either on a hydraulically bound base course or on unbound base course (crushed aggregate). These pavement structures operate without failure for 30 years and withstand 76 million ESALs.

## Figures and Tables

**Table 1:** Design mechanical characteristics of concrete used in the calculation.

Concrete Mixture Type	Concrete Mixture‘s Code	Amount of Fiber, kg/m^3^	Amount of Silica Fume, kg/m^3^	Compressive Strength, MPa	Tensile Splitting Strength, MPa	Tensile Elastic Modulus, MPa	Flexural Strength, MPa
REF w/o silica fume	Ref	0.0	0.0	41.7	2.8	51,084	5.4
REF w/silica fume	SF	0.0	25.2	57.3	3.3	60,248	7.3
Steel fiber w/o silica fume	S	49.5	0.0	52.2	5.2	83,385	7.7
Steel fiber w/silica fume	S + SF	49.5	25.2	62.3	6.0	91,428	9.0
Polypropylene_A fiber w/o silica fume	PP_A	10.0	0.0	54.5	5.6	78,346	7.3
Polypropylene_A fiber w/silica fume	PP_A + SF	10.0	25.2	59.2	5.7	75,945	8.8
Polypropylene_B fiber w/o silica fume	PP_B	15	0	56.9	5.0	83,014	7.5
Polypropylene_B fiber w/silica fume	PP_B + SF	10	25.2	59.2	5.0	73,635	8.3

**Table 2:** Design criteria for RDO Beton 09 method and design software StreetPave.

RDO Beton 09			StreetPave	
Boundary conditions	Criteria		Boundary Conditions	Criteria
	At Longitudinal Joint	At Transverse Joint		
Bearing capacity	M_R,BCBC,L_ ≥ M_E,BCBC,L_^1^	M_R,BCBC,T_ ≥ M_E,BCBC,T_	Fatigue	$\sum \frac{N_{\mathrm{design}}}{N_{\mathrm{limit}}}\leq 1.0$ ^2^
Serviceability	M_R,BCBC,L_ ≥ M_E,BCBC,L_	M_R,BCBC,T_ ≥ M_E,BCBC,T_	Erosion	$\sum \frac{N_{\mathrm{design}}}{N_{\mathrm{limit}}}\leq 1.0$
Fatigue resistance	M_R,F,L_ ≥ M_E,F,L_	M_R,F,T_ ≥ M_E,F,T_		

^1^ M_R_ is a limit bending moment, and M_E_ is acting bending moment. ^2^ N_design_ is a design load, and N_limit_ is a limit load.

**Table 3:** Input parameters for calculation according to RDO Beton 09.

Parameter		Value
Road category		National roads
Design speed		90 km/h
Traffic distribution per day		Normal
Reference axle load		70 kN
Bedding modulus, N/mm^3^	On crushed aggregate base	0.12
	On hydraulically bound base	0.15
Factor for determining the temperature gradient, –		0.14
Thermal expansion coefficient of concrete, 10^−6^/K		11.0
PCP slab dimensions, m		4.60 × 4.10

**Table 4:** Theoretical pavement structure layers below PCP slab.

Layer	Thickness, cm	E modulus, MPa
Hydraulically bound base/crushed aggregate base	15/20	10,350/310
Frost-resistant subbase	45	150
Subgrade	–	45

**Table 5:** Input parameters used in StreetPave design software.

Parameter	Value
Terminal serviceability	2
Reliability	80%
CBR	5%
Percent of slab cracked at the end of design life	15%
k	193.5 MPa/m
Flexural strength	According to Table 1

**Table 6:** Calculated thickness of PCP according to RDO Beton 09 and design program StreetPave.

Concrete Mixture Type	Unreinforced Joints				Reinforced Joints			
	On Hydraulically Bound Base		On Crushed Aggregate Base		On Hydraulically Bound Base		On Crushed Aggregate Base	
	RDO Beton 09	StreetPave	RDO Beton 09	StreetPave	RDO Beton 09	StreetPave	RDO Beton 09	StreetPave
Ref	366	246	376	251	316	211	322	214
SF	333	230	344	235	285	197	291	200
S	253	228	264	233	212	195	221	198
S + SF	229	220	240	225	192	187	200	190
PP_A	239	230	251	235	201	197	209	200
PP_A + SF	235	220	246	225	198	187	205	190
PP_B	257	230	269	235	217	197	225	200
PP_B + SF	255	225	266	230	215	192	223	195

Notes: Cells color red → the thickest PCP slab. Cells color green → the thinnest PCP slab.

## Data Availability

The data presented in this study are available on request from the corresponding author.
